# Automatic Lexical Access in Visual Modality: Eye-Tracking Evidence

**DOI:** 10.3389/fpsyg.2018.01847

**Published:** 2018-10-02

**Authors:** Ekaterina Stupina, Andriy Myachykov, Yury Shtyrov

**Affiliations:** ^1^Center for Language and Brain, National Research University Higher School of Economics, Moscow, Russia; ^2^Institute for Cognitive Neuroscience, National Research University Higher School of Economics, Moscow, Russia; ^3^Department of Psychology, Northumbria University, Newcastle upon Tyne, United Kingdom; ^4^Center of Functionally Integrative Neuroscience (CFIN), Department of Clinical Medicine, Aarhus University, Aarhus, Denmark

**Keywords:** automaticity, visual word comprehension, eye movements, parafoveal processing, visual asymmetry

## Abstract

Language processing has been suggested to be partially automatic, with some studies suggesting full automaticity and attention independence of at least early neural stages of language comprehension, in particular, lexical access. Existing neurophysiological evidence has demonstrated early lexically specific brain responses (enhanced activation for real words) to orthographic stimuli presented parafoveally even under the condition of withdrawn attention. These studies, however, did not control participants’ eye movements leaving a possibility that they may have foveated the stimuli, leading to overt processing. To address this caveat, we recorded eye movements to words, pseudowords, and non-words presented parafoveally for a short duration while participants performed a dual non-linguistic feature detection task (color combination) foveally, in the focus of their visual attention. Our results revealed very few saccades to the orthographic stimuli or even to their previous locations. However, analysis of post-experimental recall and recognition performance showed above-chance memory performance for the linguistic stimuli. These results suggest that partial lexical access may indeed take place in the presence of an unrelated demanding task and in the absence of overt attention to the linguistic stimuli. As such, our data further inform automatic and largely attention-independent theories of lexical access.

## Introduction

We use language every day to express our thoughts, emotions, and ideas to other people. It comes to us “naturally,” and we rarely think about how complex the structure and the organization of our sentences are. At the same time, language comprehension is a very complex process, involving multiple levels of processing, and it is performed in a fast and a highly efficient manner. Previous studies have shown (Shtyrov et al., 2013; Shtyrov and MacGregor, 2016) that language processing is both an extremely rapid and, at least to some extent, an automated function implying that it can be carried out even in the absence of focused attention on the input (Humphreys et al., 1982; Chiarello, 1985; Näätänen, 2001; Pickering and Garrod, 2004). This automaticity of language processing has been demonstrated at different levels of language organization: phonology (Humphreys et al., 1982), syntax (Pickering and Branigan, 1999), lexical and semantic access (Shtyrov and Pulvermüller, 2007), and even at the level of discourse (Pickering and Garrod, 2004).

Even though the term “automaticity” implies a relative degree of independence from attentional control of the linguistic input, in most reported studies, participants are in fact required to attend to at least some features of the presented linguistic stimuli. For example, participants in some studies were required to make a judgment (Maess et al., 2006; Lehtonen et al., 2011) associated with the stimulus’ familiarity or correctness or even perform a specific linguistic task (such as semantic or lexical decision, e.g., Trauer et al., 2015). As a result, it may be problematic to assert the automaticity of language processing in experimental situation when at least a degree of attention allocation is required.

On the other hand, there are a number of tasks that can be used to divert participants’ attention away from the experimental stimuli. In the auditory modality, participants are often asked to read a stimulus-unrelated text (Paavilainen et al., 1993; Muller-Gass et al., 2005), watch a silent movie or a cartoon (Leminen et al., 2012), or perform a visual detection task (Alho et al., 1994; Kathmann et al., 1999) while ignoring the auditory stimuli. The diversion task can be also presented in the same modality: e.g., a discrimination task in another auditory channel (Paavilainen et al., 1993; Alho et al., 1994). In the visual modality, attention distraction becomes particularly challenging since the very location of a stimulus within the visual field normally attracts overt attention. Two commonly used approaches to study automaticity of language processing in the visual modality are *masked priming* and the *visual Stroop* paradigms. In the masked priming paradigm (Naccache and Dehaene, 2001; Henson, 2003; Lehtonen et al., 2011), a masked “prime” stimulus is presented briefly before a “probe” stimulus, so that participants would not be consciously aware of its presence. While such studies report different priming effects including traces of lexical-semantic access (Brown and Hagoort, 1993; Kiefer, 2002), these effects are not accompanied by attentional withdrawal *per se*, but rather by reduced awareness, as participants are still required to pay close attention to the spatial location where momentary linguistic stimuli are displayed. The same applies to behavioral studies using different versions of the Stroop paradigm (Glaser and Glaser, 1982; Brown et al., 1995): even though the task does not typically encourage overt reading *per se*, word stimuli are typically presented in the focus of attention, so lexical access may occur quite explicitly despite the task requirements.

An alternative approach that would ensure attention withdrawal is to present linguistic material outside of the visual focus of attention. Existing studies using variants of this approach (Shtyrov et al., 2013; Shtyrov and MacGregor, 2016) registered early (as early as ∼70 ms) lexically specific brain responses to visual linguistic stimuli presented parafoveally, i.e., outside the attentional focus and under the condition of supposed attentional withdrawal. These became manifest as enhanced neural activity for familiar words as opposed to visually and psycholinguistically matched meaningless stimuli (“pseudowords”), which has been interpreted as automatic activation of long-term word memory traces, robust enough even when attention is diverted away from the input. These studies did not, however, control for eye movements leaving a possibility that the lexical stimuli may have been foveated and overtly processed by the participants, even though the ultra-early time-course of the neural responses suggests otherwise.

To address this caveat, we here used eye-tracking methodology in order to investigate a degree of attention allocation/automaticity during such parafoveal lexical stimulation as well as to assess spatial and lexical effects on eye movements resulting from brief unattended presentation of orthographic stimuli. For these purposes, we closely followed the experimental setup used in a previously reported EEG study (Shtyrov et al., 2013). We extended this design to control for spatial stimulus location and recorded and analyzed oculomotor activity during the task performance. Direct control of the participants’ oculomotor behavior allows for a better understanding of whether previously documented lexically specific brain responses could be registered in the absence of overt attention or could possibly be due to gaze shifts to the parafoveally presented orthographic stimuli.

Participants performed a dual feature detection (color-matching) task in the central visual field with simultaneous parafoveal brief (100 ms) presentation of three types of orthographic stimuli: *words, pseudowords*, and *non-words*. There was no task associated with orthographic stimuli and their locations varied. Participants were asked to fixate the central fixation point surrounded by two colored rings and to react to specific color combinations by providing a manual key press response. Eye movements were simultaneously recorded leading to the subsequent saccade analysis. After the experiment, participants were given recall and recognition tasks to assess the quality of memory traces for the linguistic stimuli encountered during the experiment hence signaling a degree to which they were processed by the brain. A small number of saccades to the parafoveally presented linguistic stimuli alongside a better-than-chance performance on the post experimental word recognition task would favor the hypothesis of lexical access automaticity as this would mean that participants did not need overt attention to the stimuli in order to process the linguistic stimuli to the point of later recognition. Conversely, a large number of saccades that landed on the stimuli and similarly good or better memory performance would suggest that the level of attentional load in the non-linguistic task was not sufficient and that participants did pay overt attention to the stimuli.

We also investigated potentially more fine-grained lexical effects on overt and covert attention allocation, as approximated by eye movements. The experimental materials included real words, pseudowords (word-like strings that look, read and sound like plausible real words under existing grammar and phonology constraints but do not exist in the lexicon), and non-words (textual stimuli which cannot possibly be a real word, e.g., 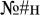). The highest level of lexicality is associated with real words, it is diminished in pseudowords, and is absent in non-words. Hence, we predicted that, if attention shifts do accompany lexical access, the associated eye movements could reveal a lexical effect: i.e., participants would be more likely to involuntarily saccade toward real words than toward pseudowords and non-words since real words are more familiar and naturally salient to the speaker (Balota et al., 2004). On the other hand, a failure to register such an effect would not necessarily imply its absence in general. Rather, it would aid in interpreting the results of the previous E/MEG experiments with experimental settings nearly identical to ours (Shtyrov et al., 2013; Shtyrov and MacGregor, 2016) and would suggest that the previously reported early lexically specific brain responses were unlikely to be driven by differential saccade artifacts for the two stimulus types.

## Materials and Methods

### Participants

Thirty-four native Russian-speaking volunteers (eight males; age range 18-27, mean 21 years old) took part in the experiment. All participants were right-handed, with normal or corrected to normal vision, and no record of neurological or language-related impairments. All participants gave informed written consent to take part in the study. The study protocol was formally approved by the HSE Ethics Committee, and participants were treated in accordance with the Declaration of Helsinki.

### Stimuli

#### Non-linguistic Primary Task Stimuli

**FIGURE 1:**
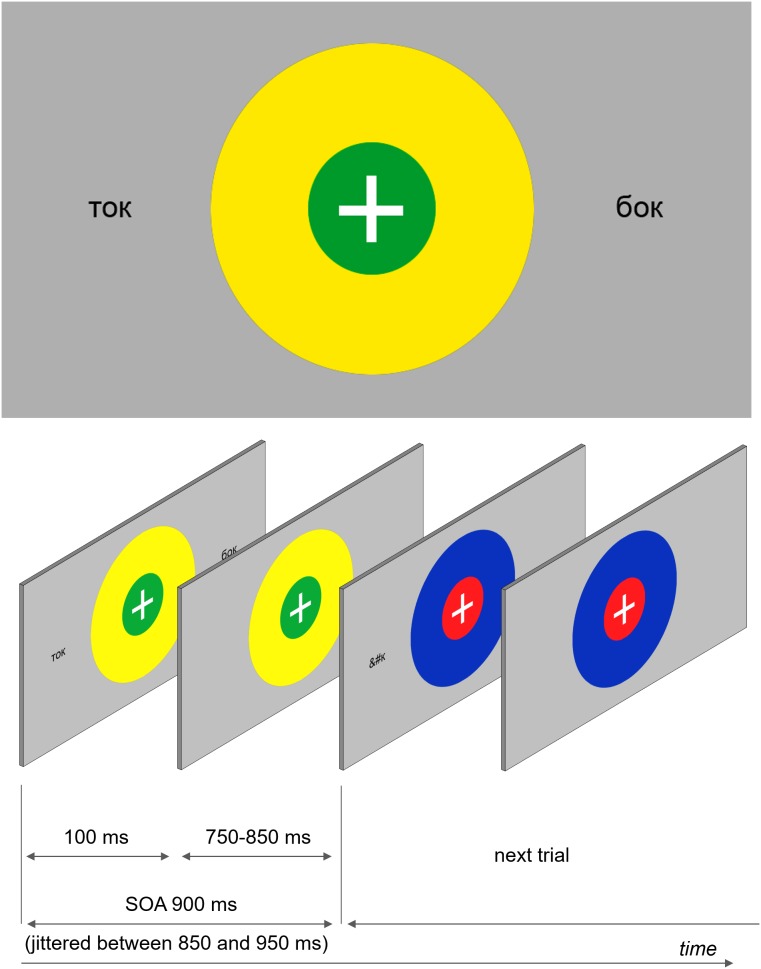
An example of a visual stimulus and a schematic demonstration of the stimulation sequence. Participants were instructed to fixate the central fixation cross presented at the center of the screen and track color combinations of two concentric circles, which were present continuously but changed colors at every SOA refresh. Orthographic stimuli were briefly (100 ms) flashed at different locations at the same time when the circles changed colors.

The main experimental task was a dual feature detection task (a color-matching task). Two concentric colored circles with a white fixation cross in the middle were presented in the center of the screen (see **Figure 1**). The outer circle’s radius was 2.5 times the radius of the inner circle (0.8° and 0.32°, respectively). All possible combinations of red, blue, yellow, and green were used. The circles were displayed on the screen for the entire duration of each trial – on average 900 ms (jittered between 850 and 950 ms).

#### Orthographic Stimuli

Here, we adopted the setup previously used to elucidate automatic visual processing of words in EEG (Shtyrov et al., 2013). We developed this protocol further in order to investigate a wider number of potentially important variables. Overall, there were four sets of orthographic stimuli. Each set consisted of three stimulus types: (1) real Russian words (e.g., 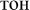 [ton] – tone; 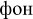 [fon] – background), (2) pseudowords (e.g., 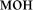 [mon]; 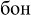 [bon]), and (3) non-words (e.g., 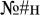; 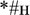). The orthographic stimuli were closely matched in a number of surface properties: (1) they were all monosyllabic three-letter strings, (2) the words and pseudowords in each set shared the last two letters and only differed in the first letter (e.g., “
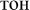
” and “
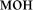
”), and (3) the combinations of the first and the last letters were counterbalanced such that they occurred an equal number of times in words and pseudowords if recombined allowing to fully exclude any low-level visual or orthographic (and related phonological) compounds.

To validate our linguistic stimulus choice, a 5-point Likert scale rating questionnaire was administered to each participant in the end of the experiment. The questionnaire included a question on lexicality of the words and pseudowords encountered by the participant during the main experiment (i.e., “*Is this a real word in the Russian language?*”). This rating study confirmed a strong word vs. pseudoword distinction as intended by the experimental setup: 4.91 word vs. 1.76 pseudowords [*F*(1,28), *F* = 5.29, *p* < 0.001, one-tailed (alpha level used throughout the paper = 0.05)].

As stated earlier, one of the objectives of the reported study was to investigate lexical effects on eye movements. In the previous experiments (Shtyrov et al., 2013; Shtyrov and MacGregor, 2016), two copies of each stimulus in each trial were simultaneously displayed at symmetrical locations at 2.5 visual degrees away from the center (on the left and on the right). In the present study, we further manipulated a number of parameters including the relative distance of the orthographic stimulus’ location from the center of the screen, the number of stimuli presented in a given trial, the types of the stimuli presented, and the arrangement of the stimuli on the screen: (1) number of stimuli displayed (one location or two symmetric locations), (2) same type or different (e.g., two copies of the same word or a word and a pseudoword), (3) distance relative to the central fixation point (2.5° or 5°), and (4) orientation of stimulus presentation (horizontally/vertically, i.e., to the left/right of the fixation point or above/below it). By adding the vertical orientation of stimulus presentation, we intended to control for the potential effects of the left-to-right reading bias.

Each condition was displayed 16 times for each of the four sets of stimuli (four words, four pseudowords, and four non-words), resulting in 64 trials per condition. Orthographic stimuli appeared on the screen simultaneously with the colored circles, but then disappeared after 100 ms, while the circles remained until the next trial. In addition, a sensory visual baseline condition was included that only contained concentric circles but no orthographic stimuli. Overall, each participant received 3904 trials; each experimental session lasted approximately 1 h and 20 min (divided into four experimental blocks with short breaks between the blocks). Trial presentation was individually randomized.

### Task and Procedure

Participants were instructed to first fixate the central fixation cross and continue to maintain this central fixation until the main task. For the main task, participants were given a target color combination (e.g., outer circle red, inner circle blue), and they were instructed to stay alert and press a joystick key with their right index finger each time they encountered this combination. The target combination featured in 15% of the trials. Participants were instructed to count the number of target combinations and report it every minute (every 60 trials). While the 15% was an average percentage of the target trials, since the trials were randomized, the number of *target* combinations each minute was not the same. Thus, participants had to count *these* to report the right number. This manipulation was introduced to make participants pay closer attention to the task. The experiment was divided into four blocks each lasting approximately 17 min. The target color combination was different in each block.

To allow participants to rest their eyes and to recalibrate the eye tracker periodically, we introduced short breaks after every 60 experimental trials, at which participants were shown four landscape pictures. They were asked to examine each picture and decide which one they liked more by pressing a designated keyboard key. These choices were not recorded. After each break, a drift check was performed, and the experiment continued when the participant refixated the central fixation point.

Participants did not receive any specific instruction about the orthographic stimuli, and they were not encouraged to look at them. Following the experiment, in a *free recall procedure*, the experimenter asked participants whether they noticed anything in addition to the colored circles. Some participants reported that they did not see anything, some recalled one or two stimuli, while others would say that they saw some letters or symbols. These answers were documented and formally analyzed (see Section “Results”).

Following this, participants were given a list of 24 words and pseudowords in a *cued recognition task*. Eight of these items had been presented earlier during the experiment (four words and four pseudowords); 16 were filler items (new stimuli that did not feature in the experiment). The filler items were monosyllabic three-letter strings similar to the actual stimuli. All of them ended with same letters “H” [n] or “K” [k] as in the main experimental stimuli but had different initial letters. Participants were asked to indicate the items they could recognize from the experiment. After this task, participants were given the rating questionnaire to validate the choice of experimental words and pseudowords as meaningful or meaningless items. Finally, the experimenter debriefed the participant and answered any questions about the study.

### Recording and Data Processing

The experiment was designed in MATLAB (Release 2014b, The MathWorks, Inc.) using the Psychophysics Toolbox extensions (Brainard, 1997; Pelli, 1997; Kleiner et al., 2007). Stimuli were displayed on a 144-Hz monitor with display resolution of 1920 × 1080 and the monitor width of 54 cm. Participants were positioned in front of the screen at a viewing distance of approximately 76 cm. Eye movements were recorded using EyeLink 1000 Plus desk-mounted eye tracker (SR Research Ltd, 2008, 2008) at 1000 Hz sampling rate.

Only the right eye was tracked for all participants. Psychophysical configuration for saccade detection was used (SR Research Ltd, 2008). Recording parse type was set to GAZE. Saccade velocity threshold was 22° per second, saccade acceleration threshold was 4000°/s^2^, and saccade motion threshold was set to 0. The data were processed and filtered before the saccade analysis. Analysis was performed using Python programming language (version 3.6.3) and the following packages: SciPy^1^ and Cili^2^. Subsequent statistical analyses were performed in R (version 3.4.0).

It is a common practice in eye-tracking studies to perform drift check before each trial. This is done in addition to calibration at the beginning of every session (SR Research Ltd, 2008). During drift check, the participant is shown a dot in the center of the screen and asked to fixate it. The trial then starts only if the error (the distance between the expected center and the recorded fixation) does not reach the specified threshold. Drift check, therefore, is used to ensure that the latest calibration parameters are still valid since they usually degrade over time due to a variety of factors (Vadillo et al., 2015). Drift check also functions as a factual “fixation cross” in order to make sure that the participant looks at the center of the screen at the beginning of each trial.

In our experiment, we could not perform drift check before each trial since there were too many trials in each experimental session to make continuous drift checking feasible (3904 trials, 850-950 ms each). Moreover, the experiment would sharply differ from the original studies where the target colored shapes were displayed continuously with only one of the colors changing. For this reason, drift check was performed every 60 trials (approximately every minute). We excluded the trials where participants did not look at the fixation cross at the beginning of a trial during preliminary examination of the individual eye movement sample reports. To this end, we detected the trials, in which participants’ gaze deviated from the central fixation point at the onset of a given trial by the distance of more than two standard deviations. Based on this procedure, we excluded five participants’ data from further analysis as these were deviating consistently from the central fixation point indicating poor engagement with the main experimental task. Data from the remaining participants were remapped with respect to the actual starting eye positions (assuming that participants were looking at the center of the screen).

Data processing also included filtering out predictive saccades that started before linguistic stimulus presentation and trials with overlapping blinks (i.e., the blinks that occurred during linguistic stimulus presentation).

Microsaccades were not included in the analysis. Although there is no full consensus on the functionally or formally determined threshold separating saccades from microsaccades (Møller et al., 2002; Kowler, 2011), the threshold of 15 min of arc was chosen based on the existing literature. The upper threshold was set to 6.5° (the farthest stimulus was presented at 5°), as saccades with larger amplitude were not of interest for our analysis. To include all relevant saccades, we chose the cut-off amplitude of 6.5 visual degrees not to leave out saccades that started, for example, on one side of the screen, crossed the midline and got close the opposite stimulus. On average, the observed mean latency for controlled saccades is around 275-350 ms and around 230 ms for reflexive saccades (Walker et al., 2000). The distribution of saccade latencies in our data was bimodal with two local maxima at 269 and 552 ms and local minimum between them at 440 ms. This local minimum was chosen as a threshold for the latency cutoff.

Finally, we determined whether each saccade was directed toward a linguistic stimulus or not. A saccade was considered stimulus-directed if it was launched in the direction of the stimulus and if it landed closer to it.

### Statistical Analysis

Linear mixed effect modeling was used for statistical analysis of the stimulus-directed saccade probabilities. The dependent variable in this analysis was the number of stimulus-directed saccades in each trial. The random effect was participant. The fixed effects were different trial characteristics that we manipulated during the experiment: (1) distance of linguistic stimuli from the center: 2.5° or 5°; (2) number of stimuli presented in the trial: one or two; (3) orientation of stimulus presentation: horizontal/vertical; (4) specific locations: left, right, up, and down (for trials with one stimulus only); and (5) stimulus lexicality: words, pseudoword, or non-word (for symmetrical trials and trials with one stimulus only). Given the dependent variable, which is based on individual trials and not the saccades (4) and (5) can be only studied in subgroups of trials. For example, in the case of stimulus lexicality in asymmetrical trials (two stimuli of different type: e.g., word and non-word) the DV (number of stimulus-directed saccades) does not show whether the saccade was made to the word or the non-word. On the other hand, for the symmetrical and single stimulus trials, we can unequivocally say what type of stimulus all stimulus-directed saccades were directed to (since they were of the same type or there was only one stimulus). Saccade latencies were examined and compared using two-sample Kolmogorov-Smirnov tests. For statistical analysis of the recognition task data, *d*′ statistics were used. For each participant, Hit and False Alarm rates were calculated. Extreme values (zeros) were adjusted to 1/(2*N*), where *N* is the number of the experimental/filler items in the list (Macmillan and Kaplan, 1985). We then tested whether the average *d*′ was significantly different from zero by a single sample *t*-test.

### RESULTS

### Non-linguistic Primary Task Accuracy

All participants successfully performed the dual feature detection task (identification of colors of the two concentric circles). Average accuracy was 98.9% (range: 94.2-99.7%).

### Recall

Following the experiment, participants were asked to recall anything apart from the colored circles. These free recall data were sparse precluding formal statistical analyses: of 29 participants, only four recalled at least one actual orthographic stimulus. However, most of the participants reported that they saw symbols, letters, or words (although they could not remember what these words were). Finally, four participants reported that they did not see anything apart from colored circles.

### Recognition

**FIGURE 2:**
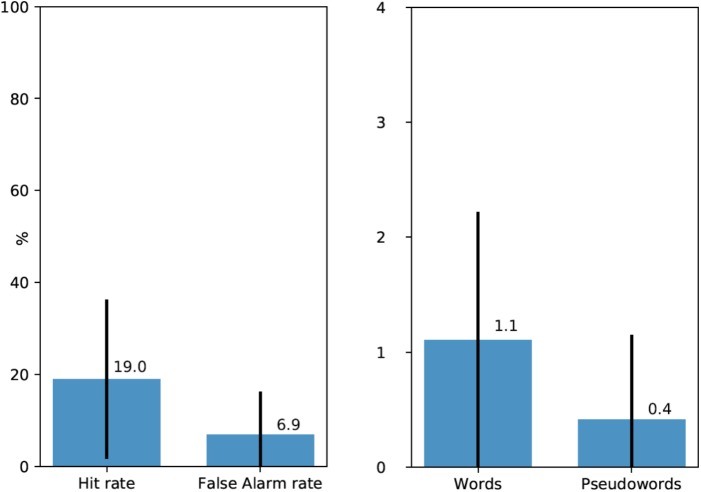
**(Left)** Average Hit rates and False Alarm rates in the recognition test (with standard deviations). **(Right)** Average number of recognized experimental words and pseudowords (out of four for each type) per participant (with standard deviations).

Following the free recall task, participants were presented with a list of words and pseudowords, eight of which they encountered earlier during the experiment (four words and four pseudowords), and 16 were new word and pseudoword foils matched in their properties with experimental stimuli (see above). 20 participants (67%) recognized at least one linguistic stimulus (*M* = 1.52 out of 8, SD = 1.38). Average Hit rate per participant was 0.19 and average False Alarm rate was 0.069 (see **Figure 2**, left). *d*′ statistics were calculated for each participant using the following formula: *z*(Hit rate) - *z*(False Alarm rate). Zeros (a situation, when a participant did not recognize any experimental stimuli or when they mistakenly chose a filler item) were adjusted to 1/(2*N*) in order to calculate *z*-transforms. For all except three participants, *d*′ values were positive (*M* = 0.6, SD = 0.48, range – from -0.48 to +1.54). The average was also significantly above zero [*t*(28) = 6.73, *p* < 0.001].

Thus, participants showed above chance performance on the recognition task. Even though most participants could not recall seeing linguistic stimuli during the experiment, they were better than chance at recognizing them when these stimuli were presented along other words in a recognition list.

Moreover, participants mostly recognized real words rather than pseudowords. 72.7% of all recognized experimental stimuli were words and only 22.3% – pseudowords. **Figure 2**, right shows the average number of recognized experimental words and pseudowords per participant: 1.1 out of four for words and 0.4 out of four for pseudowords However, data for these latter comparisons were too sparse for formal statistical analysis.

### Saccade Analysis

Overall, participants hardly ever looked at the linguistic stimuli. Only 0.004% of all saccades (413 out of total of 107,780 recorded saccades, summarized for all participants; the mean of individual percentages is also 0.004%) landed within the region of interest (ROI) defined within 1° of visual angle around the center of the linguistic stimulus (the orthographic stimuli were fully contained within the ROI). Participants directly foveated the location where linguistic stimuli were presented on average only once in 274 trials (approximately, once in every 4 min). All but nine saccades were executed after the linguistic stimuli had already disappeared (they were presented for 100 ms).

**FIGURE 3:**
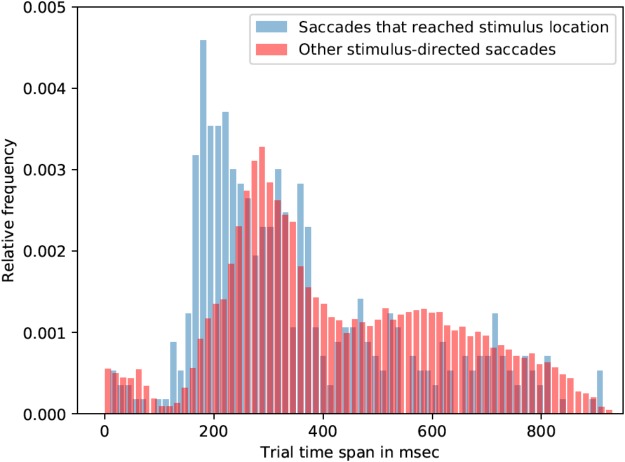
Saccade latencies for saccades that reached stimulus location and other stimulus-directed saccades (collapsed over the three stimulus types: words, pseudowords, and non-words). The histograms represent relative frequency distributions of saccade latencies (bin size = 14 ms).

Most of the saccade onsets were within the 150-400 ms range, that is, after the orthographic stimulus was already removed from the screen. Participants were seven times more likely to look on the stimulus location if the stimulus was 2.5° away from the center in contrast to 5°. Due to the lack of saccades that reached linguistic stimuli (across all conditions), all linguistic-stimulus-directed saccades were analyzed (irrespective of the type of the stimulus). On average saccades, which were directed to the linguistic stimuli, started 18 ms earlier than saccades that were not directed toward linguistic stimuli: mean latency of stimulus-directed saccades was 425 ms (std = 202 ms), while mean latency of other saccades was 443 ms. Kolmogorov-Smirnov test showed that the two distributions are significantly different (*p* < 0.001, K-S statistic = 0.05). Saccades that landed in the linguistic stimulus ROI started earlier than the rest of stimulus-directed saccades (and not directed saccades as well). Mean latency of such saccades was 355 ms (std = 202 ms), which is 71 ms earlier than other stimulus-directed saccades (see **Figure 3**) and 88 ms earlier than saccades that were not directed to the target. Kolmogorov-Smirnov test also showed that the distributions are significantly different (*p* < 0.001, K-S statistic = 0.21).

We then analyzed stimulus-directed saccades alone and investigated if the probability to make a stimulus-directed saccade depends on and can be explained by stimuli’s properties (e.g., their location, distance from the center of the screen and, most importantly, their lexicality: words vs. pseudowords/non-words).

**FIGURE 4:**
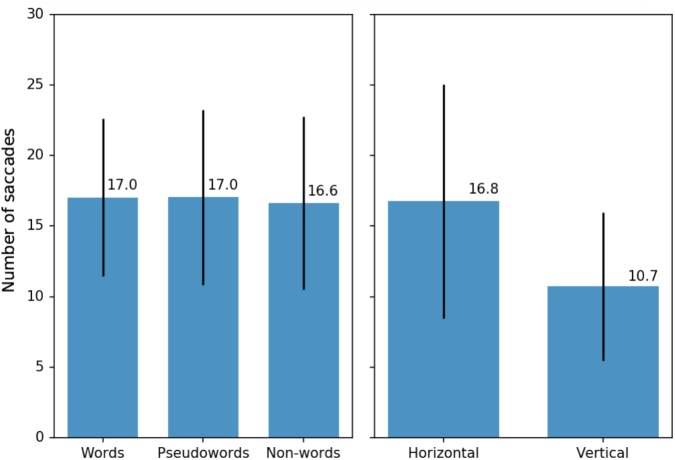
**(Left)** Average number of stimulus-directed saccades per 100 trials for three types of stimuli: words, pseudowords, and non-words (average per participant with standard deviations). Only symmetrical and unilateral trials were included in this calculation (see text for details). **(Right)** Average number of stimulus-directed saccades per 100 trials for different arrangements of the stimuli on the screen (average per participant with standard deviations). All types of trials (symmetrical, unilateral, and asymmetrical) were included in this calculation.

Linear mixed effects analysis showed no lexicality effects [χ^2^(2) = 0.534, *p* = 0.766 for the symmetrical trials and χ^2^(2) = 1.108, *p* = 0.575 for the unilateral trials] (see **Figure 4**, left). Distance from the center was only significant for the unilateral trials [χ^2^(1) = 4.529, *p* = 0.033], where distance of 5° reduced that number of stimulus-directed saccades by about six saccades per thousand trials ± 3 (standard errors). For the symmetrical trials and both the symmetrical and the unilateral trials combined, the effect of distance was not significant [χ^2^(1) = 1.632, *p* = 0.2 for the both, χ^2^(1) = 0.008, *p* = 0.931 – for the symmetrical trials]. Location of the stimulus, however, was a reliable predictor for all conditions (see in **Figure 4**, right). Vertical orientation affected the number of stimulus-directed saccades per trial [χ^2^(1) = 801.68, *p* < 0.001 for the symmetrical and the unilateral combined], reducing it by about 60 saccades per mil ± 2 (standard errors): participants were more likely to make a stimulus-directed saccade if the stimuli were presented to the left and to the right from the circle rather than above or below the circles.

## Discussion

In order to investigate automaticity in lexical access for visual linguistic information outside the focus of attention, we recorded eye movements to words, pseudowords, and non-words that were briefly presented parafoveally (at 2.5° and 5° from the center of the screen) while participants performed a dual feature (color combination) detection task. Eye movement data analysis indicated that participants very rarely saccaded to the orthographic stimuli or their previous locations. Moreover, even these few saccades did not differ between the three stimulus types. However, the post-experimental recognition analysis revealed above-chance memory performance for the linguistic stimuli suggesting that partial lexical access took place in the presence of a demanding unrelated task and in the absence of overt attention to the stimuli. We will consider these findings below.

Traditional theories of attention distinguish two modes of attentional deployment: overt and covert (Posner, 1980). Overt orienting combines shifting both attentional and visual foci to the cued location while in covert orienting these foci are diverged, and people are able to attend to a visual location without moving their eyes to it. While it is true that viewers form more detailed representations when they attend to the stimulus overtly, it is worth mentioning that attention and awareness are not the same thing, as effects of inattentional blindness, change blindness, attentional blink, among others, demonstrate dissociation between attention and awareness (Mack and Rock, 1998).

Our results demonstrate a degree of dissociation between the focus of attention and awareness. This is reflected in the sparsity of saccades in general (∼1 saccade per trial) including the extremely rare saccades that landed close to the stimulus (0.004% on average), on the one hand, and in better-than-chance post-experimental recognition of the linguistic stimuli, on the other. Indeed, post-experimental self-reports indicated that while 86% of participants noticed parafoveally presented stimuli, only 14% could recall any of them. Importantly, however, participants’ performance on a forced-choice recognition task showed a degree of word apprehension as the overall performance was above chance. Put together, these findings suggest that some linguistic information was covertly processed in the presence of an attention demanding color detection task.

However, when very infrequent saccades to the stimuli or their locations did occur, orthographic stimuli-directed saccades were on average 18 ms earlier than the undirected saccades and the saccades directed to the location of the previously presented words were even earlier – by 70 ms. This data pattern implies two things. First, it suggests that the presentation of linguistic stimuli led to partial attentional shift away from the main dual feature detection task. Second, it demonstrates that orthographic stimuli (not only words but also pseudowords and non-words) and their location facilitated this shift. The difference in saccade latencies between stimuli-directed and undirected saccades is especially intriguing if the associated attentional shifts were reflexive (involuntary) rather than controlled (voluntary). Controlled eye movements are typically slower than reflexive ones since the former have a top-down origin while the latter are under bottom-up stimuli-driven control (Walker et al., 2000). Although saccade latencies in our study were generally quite late (325 ms, following the linguistic stimuli disappearance), the fact that most of the participants (86%) reported only seeing some letters and symbols and failed to recall any of the stimuli, while the offsets and onsets of the stimuli acted as salient visual transients that tend to trigger reflexive saccades, suggests that stimuli-directed saccades were reflexive rather than controlled. It needs to be said, however, that the exact attentional mechanism underlying lexical access in our study is not completely clear. Based on the pattern described above, we propose a largely covert mechanism although it is hard to say whether such mechanism involves single or multiple foci (Posner, 1980; Scholl and Pylyshyn, 1999; Awh and Pashler, 2000).

Previous studies that manipulated attention and lexicality showed dissociations between the two. In one MEG study (Garagnani et al., 2009), participants were presented with auditory stimuli (words and pseudowords) in two conditions: under an attention-demanding task and under a distraction task. The MMN (magnetic mismatch negativity) response to words only minimally changed with attention, while the MMN for pseudowords was significantly modulated by attention, with much smaller responses in the distraction condition. The magnitude of the N400 response was also modulated by attention: in the attention condition, the responses to pseudowords were larger than to words, while the opposite was true in the ignore condition. In another study which used fMRI (Hugdahl et al., 2003), participants were presented with auditory stimuli (vowels, words, and pseudowords) under four attention conditions: passively listening to the stimuli, attending to the vowels, words, or pseudowords. The results showed distinct activation patterns depending on the attention allocation and the stimulus type.

Unlike these auditory imaging experiments and the previous EEG (Shtyrov et al., 2013) and MEG studies (Shtyrov and MacGregor, 2016) that reported early lexically specific brain responses to the linguistic stimuli presented in an experimental setting very similar to ours, we did not find any lexicality effect in the *eye movement data*. The average number of stimulus-directed saccades in a given experimental trial did not reflect the difference in the linguistic stimulus type (word, pseudoword, or non-word). That is, words did not trigger more saccades. While the absence of the lexicality effect *per se* cannot be taken as evidence of the absence of such an effect in general, it now seems unlikely that the previous E/MEG results showing rapid word-pseudoword differences in the neural activation time course were driven by any differential saccade artifacts for the two stimulus types, especially when these studies used experimental settings nearly identical to the one used in our study. This conjecture is particularly plausible if one were to take into account the fact that the earliest lexically specific E/MEG activity peaks were observed at ∼70-100 ms, whereas the average latency of saccadic shifts here was 325, implying that the automatic neural access of lexical content precedes any reallocation of attention. To fully elucidate this notion of putative automaticity in visual language processing, future studies need to combine neuroimaging and eye-tracking techniques in a single study.

There was, however, a main effect of stimulus location as there were significantly more stimulus-directed saccades when stimuli were presented to the left and to the right from the circle rather than above or below the circles. This phenomenon of horizontal bias has been well documented and investigated (Gilchrist and Harvey, 2006; Foulsham et al., 2008; Tatler and Vincent, 2008) and is likely linked to the normal reading direction in the participants’ language. This suggestion may in the future be verified by using languages with vertical writing systems.

## Conclusion

In conclusion, above-chance memory for the linguistics stimuli suggests that they must have been processed at least to some extent, while almost complete absence of saccades that landed on the stimuli and the latencies of these and other stimulus-directed saccades (which were much larger than latencies of the lexical effects observed in the E/MEG neural activations) suggest that the stimuli must have been processed covertly, thereby supporting the notion of automatic lexical access even for language presented outside the focus of attention. Future studies will need to combine both techniques in one experiment: eye movement control (eye-tracking) and neurophysiological measures (EEG and MEG) combined with neuroanatomically based current source reconstruction (using structural MRI) in order to properly delineate the time course of the neural and oculomotor responses during automatic lexical access and its neuroanatomical substrates.

## Data Availability

The raw data supporting the conclusions of this manuscript will be made available by the authors, without undue reservation, to any qualified researcher.

## Ethics Statement

This study was carried out in accordance with the recommendations of The Ethics Regulation Guidelines, The HSE Committee on Interuniversity Surveys and Ethical Assessment of Empirical Research. Ethical approval was issued by the HSE Committee on Interuniversity Surveys and Ethical Assessment of Empirical Research. All subjects gave written informed consent in accordance with the Declaration of Helsinki.

## Author Contributions

ES, AM, and YS designed and conceptualized the study. ES carried out the research and analyzed data. ES, AM, and YS wrote the manuscript and all authors reviewed the manuscript prior to submission.

## Conflict of Interest Statement

The authors declare that the research was conducted in the absence of any commercial or financial relationships that could be construed as a potential conflict of interest.

## Footnotes

1 [Software] Jones E. et al. *SciPy: Open Source Scientific Tools for Python*. Available at: http://www.scipy.org/

2 [Software] Acland B. T., and Braver T. S. *Cili (v0.5.4)*. Available at: http://doi.org/10.5281/zenodo.48843
